# Quantitative stress echocardiography in pediatric patients with type-1 diabetes mellitus

**DOI:** 10.1186/2194-7791-2-S1-A8

**Published:** 2015-07-01

**Authors:** Kai O Hensel, Franziska Grimmer, Markus Roskopf, Andreas Heusch, Andreas Jenke, Stefan Wirth

**Affiliations:** Department of Pediatrics, HELIOS Medical Center Wuppertal, Faculty of Health, Witten/Herdecke University, Wuppertal, Germany

## Aims

Type-1 diabetes mellitus (T1DM) is among the most common chronic diseases in children. Serious long-term complications include both ultra-morphologic and functional cardiac impairment. Whether these pathologic processes already take place in the early phase of T1DM yet has to be determined. Aim of this prospective, blinded, clinical study was to assess the effect of T1DM on left ventricular (LV) myocardial function in pediatric patients using quantitative stress echocardiography.

## Methods

We included 40 patients with T1DM (aged 11.53 ± 3.1 years) and 44 sex-matched healthy controls (11.43 ± 2.9 years; p = 0.91). Conventional and quantitative echocardiography was performed both at rest and during physical exercise on a bicycle. Myocardial strain and strain rate were measured using speckle tracking echocardiography.

## Results

Children with T1DM had increased myocardial contractility both at rest and at all levels of physical stress testing when compared to healthy volunteers. At rest, T1DM patients had significantly higher circumferential strain rate (-2.05 ± 0.35 s^-1^) than healthy controls (-1.86 ± 0.25 s^-1^; p = 0.016). During exercise, diabetic children showed more negative values for longitudinal strain rate (-2.59 ± 0.47 s^-1^) than healthy controls (-2.32 ± 0.41 s^-1^; p = 0.021). Furthermore, in T1DM patients, disease duration inversely correlated with myocardial strain rate and serum HbA1c levels correlated with strain both at rest and during stress testing.

## Conclusion

Long-term impairment of cardiac function in diabetic cardiomyopathy may be preceded by a transient phase of increased myocardial contractility early in the course of T1DM. The extent of hyperglycemia in childhood correlates with the alteration of myocardial contractility.

